# Impact of Video Distraction on Anxiety During Anesthesia Induction in Pediatric Patients Premedicated With Midazolam: A Randomized Controlled Trial

**DOI:** 10.1111/pan.15105

**Published:** 2025-04-03

**Authors:** Armin Sablewski, Thorben Jacobi, Sebastian Walter, Hiltrud Muhle, Christian Kandzia, Asita Fazel, Andreas Meinzer, Dithild‐Angelika Melchior, Amke Caliebe, Michael Kalab, Tobias Becher, Ingmar Lautenschläger

**Affiliations:** ^1^ Department of Anesthesiology and Intensive Care Medicine University Hospital S‐H Kiel Germany; ^2^ Department of Neuropediatrics University Hospital S‐H Kiel Germany; ^3^ Department of Ophthalmology University Hospital S‐H Kiel Germany; ^4^ Department of Otorhinolaryngology, Head and Neck Surgery University Hospital S‐H Kiel Germany; ^5^ Department of General, Visceral, Thoracic, Transplant and Pediatric Surgery University Hospital S‐H Kiel Germany; ^6^ Department of Urology University Hospital S‐H Kiel Germany; ^7^ Institute of Medical Informatics and Statistics University Hospital S‐H Kiel Germany

**Keywords:** midazolam, premedication, preoperative anxiety, video distraction

## Abstract

**Background:**

Midazolam is commonly used and effective in reducing preoperative anxiety in children. Nonpharmacological interventions, such as video distraction, are also well established for alleviating preoperative anxiety in pediatric patients, particularly those treated in ambulatory settings.

**Aims:**

To explore whether video distraction during anesthesia induction provides additional anxiety reduction in children premedicated with midazolam.

**Methods:**

In this prospective randomized controlled trial, children aged 2–10 years scheduled for elective noncardiac surgery were premedicated with midazolam and randomly assigned to either a video group (*n* = 54) or a control group (*n* = 51). In the video group, videoclips were displayed as a distraction prior to anesthesia induction, while the control group received standard care. Anxiety was measured using the short form of the modified Yale Preoperative Anxiety Scale (mYPAS‐SF). The primary endpoint was the change in mYPAS‐SF scores between transfer to the operating theater and anesthesia induction (ΔmYPAS‐SF). Secondary endpoints included emergence delirium, postoperative pain, and compliance during anesthesia induction. Character traits were assessed.

**Results:**

There was no additional anxiety reduction in the video group compared to the control group. The change in mYPAS‐SF scores (median [interquartile range]) was 4.2 (−2.1, 16.7) in the control group and 4.16 (−2.1, 7.0) in the video group (*p* = 0.246). Similarly, there were no significant differences between the groups regarding compliance during anesthesia induction, emergence delirium, or postoperative pain. Compliance during anesthesia induction, emergence delirium, and postoperative pain was similar between the groups. Additional anxiety reduction through video distraction was observed in children with pronounced anxiety traits, including “General Phobia,” “Separation,” “Panic,” and the overall “Total Phobia” score.

**Conclusion:**

In our study, video distraction did not result in additional anxiety reduction in children premedicated with midazolam prior to anesthesia induction in a hospital setting. Certain children with specific personality traits may still benefit from this intervention.

**Trial Registration:**

Registry: German Clinical Trial Register; Registration number: DRKS00025411; Principal investigator's name: Armin Sablewski; Date of registration: February 15, 2022; https://drks.de/search/en/trial/DRKS00025411

## 1 Introduction

Managing preoperative children's anxiety (POCA) is a daily challenge in pediatric anesthesia with an incidence of approximately 42%–75% [1]. Increased anxiety levels in children correlate with decreased cooperation during anesthesia induction and with increased postoperative adverse reactions such as a high rate of pain and pediatric emergence delirium [2, 3]. Therefore, it is essential to keep POCA levels low before and during anesthesia induction. Different pharmacological and non‐pharmacological treatment options for reducing POCA are available [4, 5].

As a pharmacological treatment, sedative premedication with midazolam is common and effective in reducing POCA. However, its use remains controversial because of its potential side effects, such as long sleep, amnesic effects, postoperative bradypnea, and paradoxical reactions [6, 7, 8]. Non‐pharmacological alternatives have the advantage of reducing POCA without risking these pharmacological side effects. There are a variety of distraction strategies for reducing POCA, such as parental presence, clown doctors, using virtual reality, music therapies, video games, and showing videos [9, 10, 11, 12, 13, 14].

Video distraction methods are popular due to their simplicity, widespread availability, and low costs. Individual studies have shown that video distraction may lead to anxiety reduction in non‐premedicated pediatric outpatients [15]. Another study conducted in an ambulatory setting revealed that premedicated children do not experience any additional benefits from video distraction in terms of anxiety reduction in the holding area before surgery [16]. Yet another ambulatory study demonstrated that the use of video glasses and midazolam, either alone or in combination, helps maintain baseline anxiety levels during transport to the induction of anesthesia but does not lead to their reduction [17].

It remains unclear whether these results from ambulatory settings are also transferable to the hospital setting. Our primary objective was to investigate whether video distraction in premedicated children leads to a reduction in anxiety at the time of anesthesia induction, a period when children's anxiety levels typically peak [5]. As a secondary objective, we tested the hypotheses that using video distraction reduces the incidence of emergence delirium and postoperative pain and improves children's compliance during anesthesia induction. Furthermore, we investigated exploratively whether personality traits are related to the success of video distraction.

## 2 Methods

This monocentric randomized controlled trial was approved by the local Institutional Review Board (Ethics Committee of Christian‐Albrechts‐University of Kiel, Germany, ethics approval number D418/21) and registered at the German Clinical Trials Registry (date of first trial registration February 15, 2022, registration number DRKS00025411, https://drks.de/search/en/trial/DRKS00025411). The first patient was recruited on April 1, 2022. Written informed consent was obtained from the parents or legal guardians of the minor subjects. This manuscript adheres to the applicable CONSORT guidelines. The study was conducted at the University Hospital Schleswig‐Holstein in Kiel, Germany, between March 2022 and March 2023. This study was performed in accordance with the relevant guidelines and regulations as well as in accordance with the Declaration of Helsinki.

### 2.1 Study Participants

We included hospitalized children aged 2–10 years who were scheduled for elective noncardiac surgery under general anesthesia. Participants were included in the study after obtaining informed consent from both parents and, when feasible, from the children themselves, as part of the standard daily procedure. Children with limitations in communication such as severe deafness, severe visual impairment, or mental disability were excluded, as well as children who had undergone general anesthesia in the last 6 months and those with contraindications for midazolam and/or EMLA (eutectic mixture of lidocaine and prilocain).

### 2.2 Measures

The short form of the Modified Yale Preoperative Anxiety Scale (mYPAS‐SF) was used to assess children's anxiety on the day of surgery. The mYPAS‐SF is an observational measure of preoperative anxiety consisting of 18 items in four domains (activity, emotional expressivity, state of arousal, and vocalization). The mYPAS‐SF total score ranges from 22.91 to 100, with higher scores indicating greater anxiety. Scores above 30 are typically considered indicative of the presence of anxiety. Two observers were trained to assess the mYPAS‐SF, and at least one of them rated each child's anxiety.

A visual analog scale (VAS) was used to measure children's anxiety (VAS‐anxiety) and pain (VAS‐pain) by self‐assessment. These scales range from 0 (no fear/pain) to 10 (maximum fear/pain) [18, 19]. For children up to 4 years of age, we instead used an external assessment scale (the Children's and Infants' Postoperative Pain Scale, CHIPPS) to measure pain before and after surgery. This scale contains 5 domains (crying, facial expression, body posture, leg posture, and motoric activity) with 3 levels (each 0, 1, or 2 points) ranging from 0 (no pain) to 10 (maximum pain) [20].

Children's compliance with the induction of anesthesia was measured using the Induction Compliance Checklist (ICC). The ICC represents the sum of the 10 negative behavioral categories evaluated during induction, with higher scores indicating lower behavioral compliance [21, 22].

Postoperative pediatric emergence delirium was scored using the Pediatric Anesthesia Emergence Delirium (PAED) scale by Sikich and Lerman to assess the severity of children's gesturing in the recovery room [23, 24]. Levels greater than 9 indicated the presence of agitation and the need for treatment.

To assess personality traits, we utilized standardized validated questionnaires that were completed by parents during the hospital stay. Symptoms of anxiety disorders were evaluated using diagnostic criteria from the mental disorder classification systems ICD‐10 and DSM‐5 for children and adolescents, as outlined by Döpfner et al. [25]. The children's temperament was assessed using the Inventory for Temperament in Children (IKT) via a parent‐reported questionnaire comprising five items: susceptibility to frustration, inhibition, activity level, perseverance/attention, and sensory sensitivity [26]. Conduct problems were evaluated using the Strengths and Difficulties Questionnaire (SDQ) [27].

### 2.3 Study Design

Information about anesthesia and details of the study were provided to the children and their caregivers during the pre‐surgery anesthesia consultation. An anesthesiologist informed all families in detail and in a child‐friendly manner about the anesthesiologic procedures and the study. Two EMLA patches were handed out and instructions for their use were given. According to the manufacturer, the patches require a minimum of 60–90 min of exposure time and should be removed 20–40 min before venipuncture.

**FIGURE 1:**
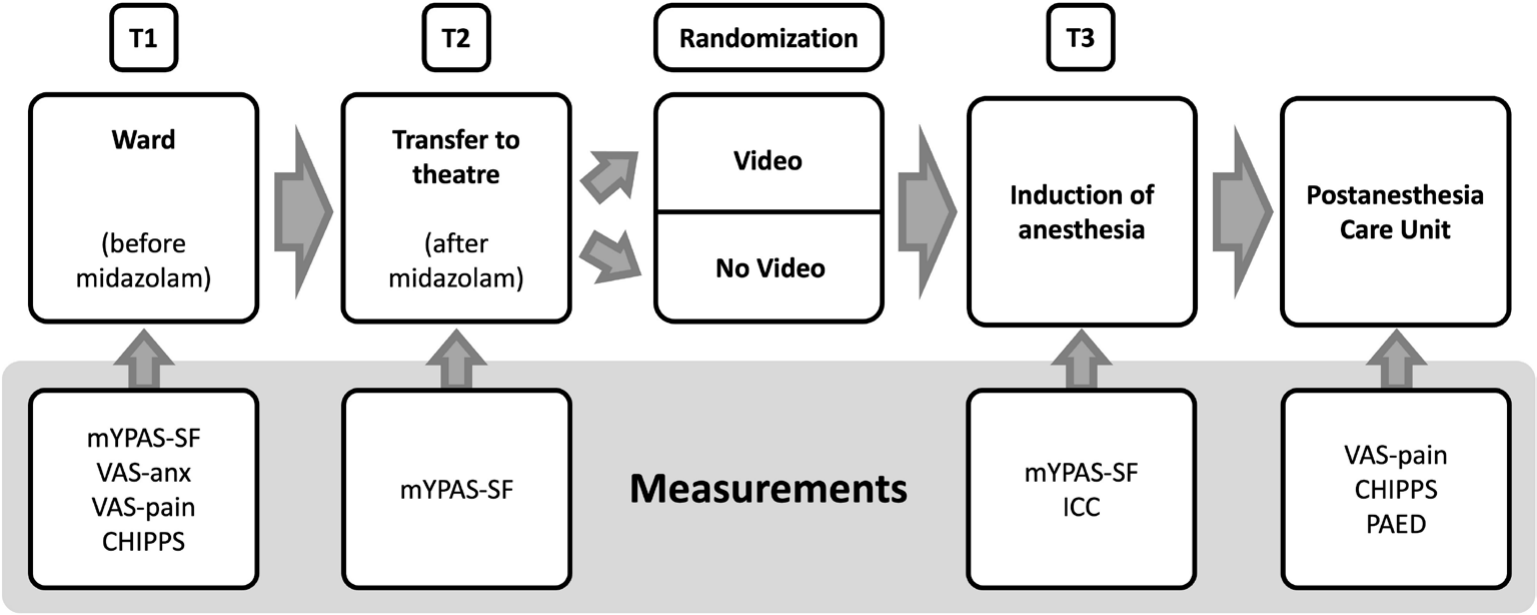
Schematic workflow and measurements recorded through the study. CHIPPS, Children's and Infants' Postoperative Pain Scale; ICC, Induction Compliance Checklist; mYPAS‐SF, Modified Yale Preoperative Anxiety Scale—Short Form; VAS‐anx, Visual analog anxiety scale; VAS‐pain, Visual analog pain scale.

On the day of surgery, all children underwent a standardized perioperative procedure (Figure 1). Upon arrival at the ward (T1), the child's anxiety level was assessed using the mYPAS‐SF, and pain and anxiety levels (VAS‐pain and VAS‐anx at T1) were recorded for cooperative children. For children up to 4 years old, pain levels were assessed using the CHIPPS.

Midazolam was administered for premedication either orally at a dose of 0.5 mg/kg body weight or rectally at a dose of 1 mg/kg body weight (up to a maximum of 15 mg, tolerance range ± 20%) approximately 20–40 min before the expected induction of anesthesia. After the administration of midazolam, the EMLA patches were removed. The administration time of midazolam was documented to ensure consistent timing.

The children were transferred from their hospital beds to the operating theater (transfer to theater = T2). Here, anxiety levels were reassessed using the mYPAS‐SF after the full effect of midazolam was expected, as it had been administered 20–40 min earlier. Following this assessment, the children were separated from their parents and randomized into either the control or video group.

The randomization for the study was conducted using the website www.randomizer.org. Earlier, a neutral person prepared sealed envelopes labeled “Control” or “Video” based on the generated numbers. These envelopes were stored at the Anesthesiology and Operative Intensive Care Clinic at UKSH Campus Kiel and were distributed in the generated numerical order on the study day by members of the study team. In the video group, the videos were displayed on an 8‐in. tablet (SAMSUNG Galaxy Table A 8.0 Wi‐Fi (2019), 8.0 in., black) attached to a holder (Photecs Universal Tablet Holder Pro V3, attachment & holder for iPad Pro 12.9″ or other tablet PCs from 6 to 14 in.) on the surgical stretcher. The tablet was positioned comfortably for viewing videos. The videos of the child's choice were presented via Youtube or, if the child was unsure, a child‐friendly video was shown at the discretion of the supervising anesthesiologist.

### 2.4 Anesthesia Protocol

In the operating theater, standard monitoring was applied, consisting of noninvasive blood pressure measurements, pulse oximetry, and 5‐lead ECG. General anesthesia was induced either by inhalation of sevoflurane or by intravenous application of propofol at the discretion of the anesthesiologist. Intravenous access was limited to two attempts within the EMLA‐anesthetized area. A mask was gently held in front of the child's face for preoxygenation (induction of anesthesia = T3); the anxiety level was reassessed, and the ICC was recorded. After loss of consciousness, the video was stopped and the time of video distraction was recorded. The induction of anesthesia was performed by a board‐certified anesthesiologist or under their supervision, ensuring a child‐friendly approach and empathetic care within a supportive environment.

General anesthesia was maintained with continuous application of propofol (3–12 mg/kg of body weight per hour) and remifentanil (0.2–0.5 μg/kg of body weight per minute). Patients received a standard analgesic regimen with local anesthetics and were administered ibuprofen, paracetamol, and/or metamizole as needed, considering the extent of surgery and contraindications. Dexamethasone, ondansetron, and/or dimenhydrinate were administered as prophylaxis to reduce the incidence of postoperative vomiting. After surgery, the children were transferred to the postanesthesia care unit (PACU). Pain was managed by standardized medication (piritramide 0.05–0.1 mg/kg of body weight). The presence of pediatric delirium was assessed 5 min after obtaining cooperativity. The children's pain was measured using the VAS or CHIPPS.

### 2.5 Statistical Analysis

Our primary objective was to investigate whether video distraction in premedicated children would lead to an additional reduction in anxiety at the time of anesthesia induction as compared to no video distraction. The primary endpoint was the change in children's anxiety at timepoint T3 in comparison to that at timepoint T2 (Delta T3–T2). Negative values represent a decrease in the child's anxiety from T2 to T3.

The sample size calculation was performed based on data published previously by Mifflin et al., who reported an effect size of approximately *d* = 0.6 (Cohen's *d*) assuming a 15‐point difference between the groups on the mYPAS score [12]. For a power of 0.8 and a significance level of 0.05, the resulting sample size per group was at least 44 patients (two‐sided Wilcoxon rank‐sum test). A difference of 8 points between groups would be considered clinically relevant because our population was premedicated. We adjusted the sample size by +20% to mitigate the impact of possible dropouts, which resulted in a total case number of 106 patients. Sample size calculations were performed with the statistical software G*Power, version 3.1.9.6.

Secondary endpoints were the PAED score, the VAS/CHIPPS score, and the ICC score in the recovery room. Exploratory endpoints were the anxiety assessment by Döpfner et al. and the IKT and SDQ questionnaires. A subgroup analysis was conducted for the 2–5 and 6–10 age groups to display anxiety levels within each subgroup.

In the descriptive analysis, we presented absolute and relative frequencies for the respective groups for categorical variables and medians and interquartile ranges [25th–75th percentiles] for the respective groups for continuous variables. Since the continuous variables showed deviations from the normal distribution, we applied a Wilcoxon rank‐sum test for the comparison of the control and video groups with respect to these variables. Categorical variables were compared between groups using Fisher's exact test. We applied a significance level of 0.05 for all the statistical tests. All tests were two‐sided. R version 4.3.1 was used with R studio on a Fedora 39 operating system [28]. We calculated the intraclass correlation within patients who were rated by two raters to assess interrater reproducibility. To perform intraclass correlation, we used the command ICC with the parameters model = two way, type = agreement, and unit = single in the R package irr [29]. In the analysis of personality traits, children were divided into two groups with respect to the median of the trait. Delta T3–T2 was then compared between the video and control groups in the respective strata.

The primary analysis population was the full‐analysis set. As a sensitivity analysis, the per‐protocol set was used.

## 3 Results

**FIGURE 2:**
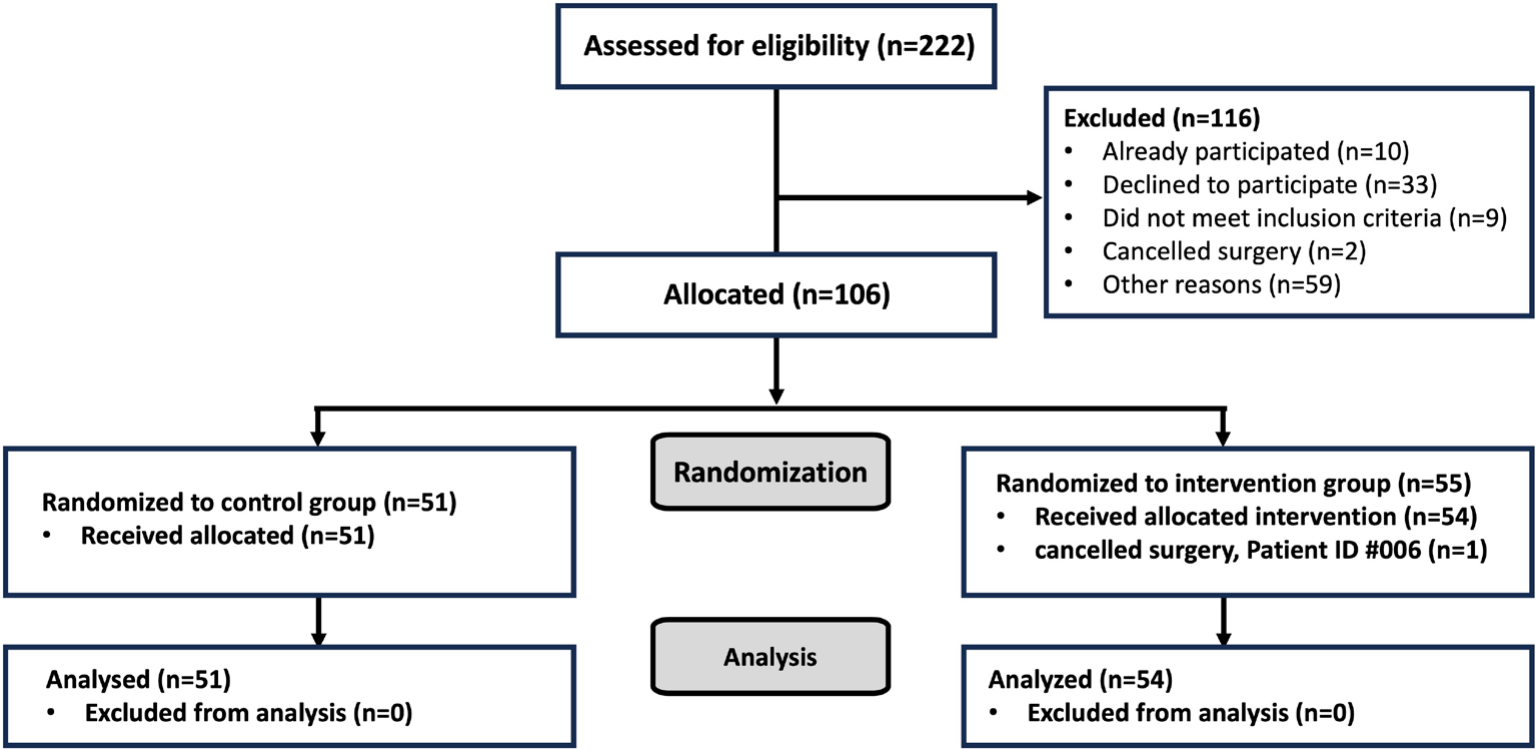
Participant workflow diagram for full‐analysis set.

A total of 106 patients aged between 2 and 10 years were enrolled (55 in the video group and 51 in the control group). One patient was excluded because no data for mYPAS‐SF T2 and mYPAS‐SF T3 could be obtained (surgery was canceled because of positive COVID‐19 testing). Therefore, the full analysis set (the primary analysis population) consisted of 105 patients (see Figure 2). If not stated otherwise, data and results refer to the full analysis set. The baseline characteristics of patients are shown in Table 1, and anesthesia settings are shown in Table 2. There were no significant differences between the control group and the video group before randomization.

**TABLE 1:** Patients' characteristics.

Variables	Control (*n* = 51)	Video (*n* = 54)
Age, months	68 [40–86]	67 [39–91]
Body weight, kg	19 [14–28]	20 [15–23]
Sex, F:M, %	49:51	43:57
ASA		
1	35 (69%)	32 (59%)
2	14 (27%)	18 (33%)
3	2 (4%)	3 (5%)
4	0	1 (2%)
Previous illness, yes	19 (37%)	30 (56%)
Previous anesthesia, yes	30 (59%)	24 (44%)
Accompanied by		
Both	5 (10%)	6 (11%)
Mother	38 (74%)	36 (67%)
Father	8 (15%)	12 (22%)

*Note:* Values are median [25th–75th] or number (proportion).

Abbreviations: ASA, physical status classification; F, female; M, male; *n*, number of patients.

**TABLE 2:** Anesthesia setting.

Variables	Control (*n* = 51)	Video (*n* = 54)
Midazolam dose, mg	10 [7.5–12]	10 [8–12]
NA	1	0
Midazolam applied, oral:rectal, %	98:2	89:11
Administration time of midazolam at T2, min	34 [24–46]	30 [23–44]
NA	1	0
Time of video distraction, min	—	11 [9–14]
EMLA patch time, min	118 [77–173]	106 [69–150]
NA	1	0
Department		
ENT surgery	13 (25%)	21 (39%)
General surgery	4 (8%)	2 (4%)
Ophthalmic surgery	28 (55%)	26 (48%)
Urological surgery	6 (12%)	5 (9%)
Anesthesia induction		
iv line 'in situ', iv induction	3 (6%)	2 (4%)
iv line established, iv induction	23 (45%)	29 (54%)
iv access frustrated, gas induction	19 (37%)	15 (28%)
Primary no iv access, gas induction	6 (12%)	8 (15%)
Airway management		
LMA	38 (75%)	38 (70%)
Mask	1 (1%)	0
Tube	12 (24%)	16 (30%)

*Note:* Values are median [25th–75th] or number (proportion).

Abbreviations: EMLA, eutectic mixture of local anesthetics, patch for local dermal anesthesia; ENT, ear nose throat; iv, intravenous; LMA, laryngeal mask; *n*, number of patients; NA, not applicable; T2, baseline at holding area after premedication with midazolam and separation from caregivers.

The per‐protocol set included all randomized patients without major protocol deviations. A total of 11 patients were excluded due to major protocol deviations (see Table S1), so that the per‐protocol set consisted of 95 patients. The trial ended according to plan after the recruitment of the planned number of cases in March 2023.

### 3.1 Children's Anxiety

The overall baseline anxiety of the children at T1 was measured with a median mYPAS‐SF score of 33.3 [22.9–39.6] and a VAS‐anxiety score of 2.0 [0.0–4.0], with no significant differences between the groups.

**FIGURE 3:**
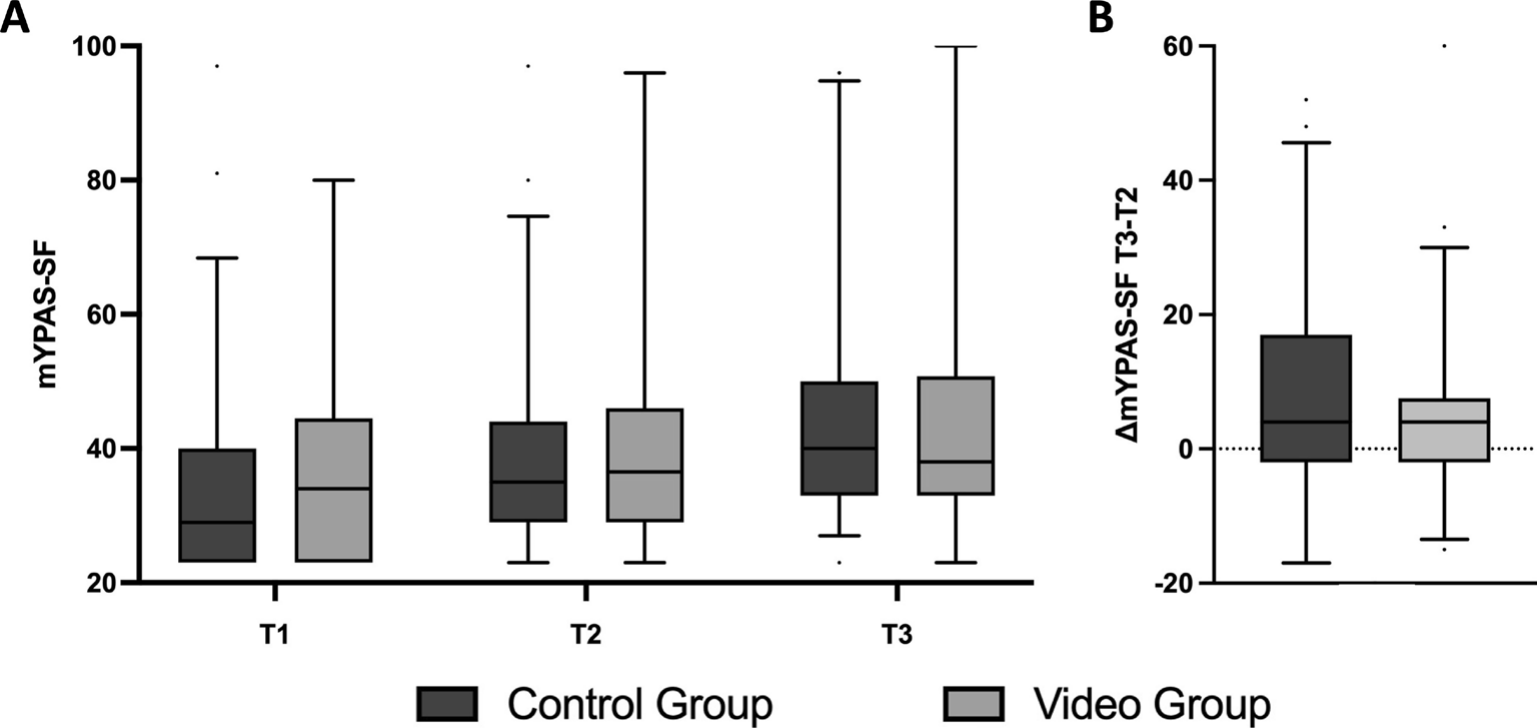
Progression of anxiety over time (A) and difference in anxiety between T2 and T3 (B). Boxes show median and IQR; whiskers 5–95 percentile range, outliers as dots; (A) showing the progression of anxiety from T1 = at the ward, T2 = while transfer to theater and T3 = during induction of anesthesia measured by mYPAS‐SF in control group and video group; (B) shows the difference between the anxiety at T3 and T2 measured by mYPAS‐SF; positive scores indicate an increase and negative scores a decrease in the children's anxiety.

At T2 (before the intervention), the anxiety measured with the mYPAS‐SF was 35.4 [29.0–42.2] in the control group and 36.5 [29.7–45.3] in the video group. At T3, there was an increase in anxiety to a median of 39.6 [33.3–50.0] in the control group and 37.5 [33.3–49.2] in the video group, but there was no significant difference (*p* = 0.363) between the groups (Figure 3A).

Regarding the primary endpoint, there was an increase in anxiety between T2 and T3 of 4.2 [−2.1 to 16.7] in the control group and 4.2 [−2.1 to 7.0] in the video group, with no significant difference between the groups (*p* = 0.246, Figure 3B). In the per‐protocol set, we also found no significant reduction in anxiety as a result of video presentation (*p* = 0.185).

Regarding the interrater agreement between the two raters for the three timepoints, we found intraclass correlation coefficients of 0.986 (timepoint T1, *n* = 52), 0.981 (T2, *n* = 50) and 0.988 (T3, *n* = 50).

### 3.2 Secondary Endpoints

The distribution of ICC scores was as follows: In the control group, 23 participants (45%) had a perfect score (ICC = 0), while 28 (54%) had not (ICC > 0). In the video group, 33 participants (61%) had a perfect score, while 21 (39%) had not. The difference in ICC scores between the groups was not statistically significant (*p* < 0.120).

For the PAED emergence delirium score, the median score for the total group was 5 [2–8], with the control group having a median of 5 [2–9] and the video group a median of 4 [2–8]. The difference in PAED scores between the groups was not statistically significant (*p* = 0.749).

In terms of postoperative pain, as measured by the VAS‐pain/CHIPPS, the total group had a median score of 3.0 [0.0–6.8], with the control group scoring a median of 4.0 [0.0–7.5] and the video group scoring a median of 2.0 [0.0–6.0]. The difference in VAS‐pain/CHIPPS scores between the groups was not statistically significant (*p* = 0.244).

### 3.3 Exploratory Endpoints

**FIGURE 4:**
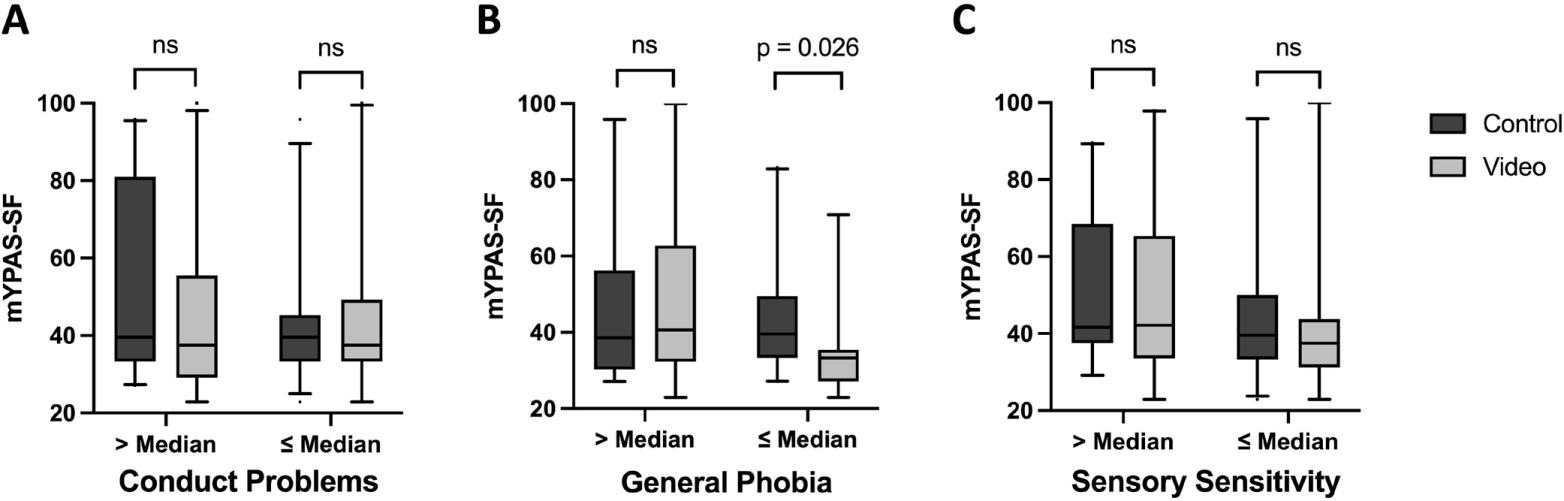
The impact of personality traits on anxiety during induction of anesthesia. Anxiety measured with mYPAS‐SF at T3 (induction of anesthesia) in control and video group with reference to personality traits. Boxes show median and IQR; whiskers 5–95 percentile range, outliers as spots. Figures show children above or below the median values of their respective raw values for personality traits. (A) Conduct problems expressed with total difficulty score of the SDQ; (B) personality trait “general phobia” of anxiety disorders; (C) personality trait of temperament with item “sensory sensitivity” of IKT.

In this study, an equal distribution of children's personality traits was observed in both groups (see Table S2). Children with less general phobia experienced an additional anxiety reduction through video distraction, while those with conduct problems or sensory sensitivity did not (Figure 4). Additional reductions in anxiety were observed in children exhibiting pronounced anxiety traits, specifically in the domains of “Separation”, “Panic”, and the overall “Total Phobia” score (a complete evaluation of all personality traits is presented in Tables S3–S5).

In the subgroup analysis based on age stratification, a baseline anxiety level at T1 of 35.4 [22.9–45.8] was observed for children aged 2–5 years (*n* = 41). For children aged 6–10 years (*n* = 64), baseline anxiety at T1 was 29.2 [22.9, 38.0]. In the younger age group, the median increase in anxiety between T2 and T3 was 10.4 [0.8, 21.1] in the control group, whereas in the video group an increase of 6.3 [0–16.7] occurred (*p* = 0.396). In the older age group, a median increase in anxiety of 1.0 [−2.6 to 11.5] was noted in the control group, while no change in anxiety was observed in the video group (0, [−6.3 to 6.3] between T2 and T3 (*p* = 0.348). A complete subgroup analysis is presented in Table S6.

## 4 Discussion

In this randomized controlled trial, no significant difference in anxiety reduction was observed between the control group and the video intervention group in children aged 2–10 years. All children were premedicated with midazolam and were hospitalized.

Our results differ from the findings of Mifflin et al., who showed that video distraction leads to a reduction in anxiety for non‐premedicated children for ambulatory surgery [15]. However, our results are in line with the data published by Sola et al. and Kerimoglu et al., who reported no additional benefit of video distraction for premedicated children in ambulatory surgery units [16, 17]. In the study by Sola et al., an anxiety baseline of 23.3 was measured using mYPAS in an ambulatory setting before administering midazolam. However, in a hospital setting, the baseline anxiety level appears to be greater, as indicated by the study conducted by Kain et al., which reported a mYPAS score of approximately 30, and Berghmans' study, which recorded a baseline score of approximately 40. Since the mYPAS‐SF was used in our study, the results are comparable to those of the aforementioned studies performed with hospitalized patients, suggesting that higher levels of anxiety were already present in the ward.

Therefore, the findings from an ambulatory setting seem not to apply to a possibly more unsettled and less child‐friendly hospital setting. The finding that video distraction did not reduce anxiety in our hospitalized pediatric patients could be due to the already distracting nature of the hospital. Additionally, factors such as a child‐friendly atmosphere, skilled anesthesia teams, and the presence or separation of parents are crucial, while video distraction is only one contributing factor and may be outweighed by other influences.

Regarding the secondary endpoints, including the reduction in pain measured with VAS/CHIPPS, emergence delirium, and ICC, we found no differences between the two groups. Interestingly, other studies have demonstrated reduced delirium rates and less need for pain medication when video distraction was offered [30, 31]. Consequently, it remains unclear which factors predominantly influenced these results.

One important question addressed in this study was whether additional anxiety reduction could be achieved with video distraction following midazolam administration. It could be hypothesized that further anxiety reduction through videos might be limited by the effects of midazolam, given its amnesic properties [32]. We observed many children treated with midazolam who still exhibited high levels of anxiety (> 40 mYPAS‐SF). This does not necessarily question the overall efficacy of midazolam premedication but rather raises the question of which children truly benefit from such premedication.

We tested in an exploratory character an individualized approach to the impact of personality traits on anxiety through video distraction. Previous studies have shown that increased temperament and increased state anxiety are predictors for higher anxiety in children during anesthesia induction [33, 34]. It was interesting to observe in our study that certain personality traits, such as lowered social phobia as a subset of anxiety character trait, may indicate that targeted interventions are particularly effective. However, this study was not designed to evaluate this aspect. Thus, further research is needed to determine which children benefit most from video distraction, if any, taking individual aspects such as personality traits and previous experiences into account.

Despite its simplicity, video distraction is not a one‐size‐fits‐all solution and requires critical evaluation. It can be implemented in various ways, with practical considerations playing a key role in its application. We opted for tablet‐based distraction, as it is widely used in clinical settings and allows for continued interaction [12]. Other common methods include smartphone‐based distraction and the use of video glasses [17]. Recent studies have shown that 3D distraction using VR glasses is equivalent to 2D distraction in terms of effectiveness but does not further reduce anxiety [13]. However, video glasses are only suitable for older children, making them less applicable for younger children, who are at higher risk for POCA. Additionally, they limit interaction, which remains possible with tablets. This enables anesthesiologists to immerse themselves in the “cartoon world” alongside the child, potentially strengthening their bond. In certain scenarios, videos might even be counterproductive, particularly if their content is overly lively and unsettling. Once again, individual personality traits may play an important role.

Several important conceptual and methodological issues and limitations should be noted.

As a video demonstration with a tablet can hardly be blinded, the observers could not be blinded either. In this study, however, two trained observers with a high interrater correlation collected the data without interfering in the anesthesiologic treatment.

There was heterogeneity in the age of the children. The potential variability in neurological maturity within the analyzed age range can affect the generalizability of the results. Children aged 2–5 years are at a greater risk of POCA, while older children can cope better with perioperative stressors [35]. Our subgroup analysis supports this observation, showing an increase in anxiety between T2 and T3 among younger patients (ages 2–5) and stable levels among older children (ages 6–10), with both groups unaffected by video distraction. These findings suggest that video distraction may not further reduce anxiety when midazolam is administered, regardless of the child's age and despite differences in anxiety progression between younger and older children. However, validation in a larger follow‐up study with an adapted study population is needed, as the sample size was not designed for this subgroup analysis.

Although the dosage of midazolam was standardized based on body weight, this may not provide conclusive insights into its effectiveness, as much as the requirement for midazolam may vary across different age groups of children. Furthermore, including a third group (video distraction only) might have enhanced the study results.

Another limitation is that the VAS‐pain score and the CHIPPS score were evaluated together. Both scales range from 0 to 10 but are conceptually different. Furthermore, although the VAS‐anx has been evaluated only for children aged 7 years and older, it was used in this study based on the child's cooperativeness, but regardless of age. These parameters had no influence on the result of the primary endpoint. Importantly, the mYPAS‐SF was not designed to assess distraction techniques, as these directly influence one of the score's domains, potentially compromising its reliability in this setting. Moreover, factors such as mental overload and periprocedural pain can also affect children's cooperativity during anesthesia induction, further interfering with the accuracy of mYPAS‐SF scoring. Nevertheless, it remains a commonly used tool in this context.

Similar to previous studies, the types of surgical procedures in this study were heterogeneous, which introduces potentially large variation [15, 16, 35]. Since the primary endpoint was measured at the induction of anesthesia prior to the start of surgery, the type of procedure likely had minimal impact on this outcome, although this possibility cannot be entirely excluded. Additionally, we did not standardize the method of anesthesia induction (whether inhalational or intravenous). Such a standardization would improve the interpretation of results, but there is the risk that such an approach may not fully reflect everyday clinical practice and can raise ethical concerns.

In accordance with standard practice in our hospitals, parents did not accompany children beyond the preoperative holding area. This approach aligns with the procedures described in the studies by Kerimoglu and Sola but differs from Mifflin, where parental presence was optional [15, 16, 17]. Parents may feel a sense of relief when accompanying their children during the induction of anesthesia, even though not all studies have demonstrated a significant reduction in children's anxiety. Additionally, another study showed that mothers who actively participated in the perioperative process—such as recording their voice for sedation during a catheterization procedure—perceived their involvement positively, which was associated with reduced pain levels in their children [36]. These findings highlight the importance of further investigating the optimal balance between distraction techniques and parental involvement during the perioperative process to improve outcomes and experiences for both children and their parents.

In conclusion, our study revealed that video distraction during the induction of anesthesia in hospitalized pediatric patients premedicated with midazolam did not lead to further anxiety reduction. There was also no additional benefit observed in postoperative outcomes such as pain or delirium rates. The investigation of certain personality traits suggests that children's anxiety could potentially be reduced through distraction techniques for particular children. Future research should focus on exploring and implementing individualized approaches to reduce anxiety in children. It will be important to determine the most effective distraction strategies and identify the specific circumstances within the hospital environment that contribute to effective anxiety reduction in children.

## Author Contributions

**Armin Sablewski:** conceptualization, methodology, validation, formal analysis, investigation, resources, data curation, visualization, writing – original draft, writing – review and editing, project administration. **Thorben Jacobi:** formal analysis, investigation, resources, data curation, writing – review and editing. **Sebastian Walter:** formal analysis, investigation, resources, data curation, writing – review and editing. **Hiltrud Muhle:** writing – original draft, writing – review and editing. **Christian Kandzia:** writing – original draft, writing – review and editing. **Asita Fazel:** writing – original draft, writing – review and editing. **Andreas Meinzer:** writing – original draft, writing – review and editing. **Dithild‐Angelika Melchior:** writing – original draft, writing – review and editing. **Amke Caliebe:** formal analysis, investigation, resources, data curation, writing – original draft, writing – review and editing. **Michael Kalab:** formal analysis, investigation, resources, data curation, writing – original draft, writing – review and editing. **Tobias Becher:** writing – original draft, writing – review and editing. **Ingmar Lautenschläger:** conceptualization, methodology, validation, formal analysis, writing – original draft, writing – review and editing, project administration.

## Ethics Statement

This trial was approved by the local Institutional Review Board (Ethics Committee of Christian‐Albrechts‐University of Kiel, Germany, ethics approval number D418/21) and performed in accordance with the relevant guidelines and regulations as well as in accordance with the Declaration of Helsinki.

## Consent

Informed consent was obtained from all parents or legal guardians of participants involved in the study.

## Conflicts of Interest

The authors declare no conflicts of interest.

## Supporting information


Data S1.

## Data Availability

The authors confirm that the data supporting the findings of this study are available within the article (and/or) its Supporting Information.
