# Supplementary material for: Impact of Video Distraction on Anxiety During Anesthesia Induction in Pediatric Patients Premedicated With Midazolam: A Randomized Controlled Trial
Source: Paediatr Anaesth. 2025 Apr 3;35(7):542–51. doi: 10.1111/pan.15105 (PMC12149489; doi:10.1111/pan.15105)
Supplement: Supplementary file 1 — Data S1. [file PAN-35-542-s001.docx]

| Patient ID | Protocol violation | mYPAS-SF T1 | mYPAS-SF T2 | mYPAS-SF T3 |
| --- | --- | --- | --- | --- |
| #001 | Midazolam dose too low | 26 | 38 | 67 |
| #004 | Midazolam dose too low | 23 | 40 | 31 |
| #006 | Randomization before the discovery of a past COVID infection within the last weeks, cancellation of surgery | 29 | 0 | 0 |
| #019 | Mother called in for induction, failure of midazolam and video therapy | 40 | 96 | 96 |
| #011 | Mother called in for induction | 37 | 33 | 33 |
| #017 | Mother called in for induction | 33 | 35 | 44 |
| #024 | Nintendo game console while transfer to operation room | 23 | 97 | 94 |
| #027 | Long waiting time until operation, repeated medication before operation | 40 | 40 | 44 |
| #044 | Mother called in for induction | 23 | 29 | 50 |
| #059 | Long waiting time until operation, repeated medication before operation | 23 | 41 | 38 |
| #093 | Mother called in for induction | 49 | 46 | 61 |

**Supplementary Table S1.** Listing of patients with protocol deviations. T1 = at the ward, T2 = while transfer to theatre and T3 = during induction of anesthesia

| Personality trait ‘anxiety’ as outlined by Döpfner and Görtz-Dorten for age ≥4 years | | | | |
| --- | --- | --- | --- | --- |
| Category | Total  (n=72) | Control Group (n=36) | Video Group (n=36) | P value |
| Separation anxiety  NA | 0.30 (0.10, 0.70) 1 | 0.20 (0.10, 0.53) 0 | 0.40 (0.20, 0.70) 1 | 0.106 |
| Panic  NA | 0.00 (0.00, 0.13)  1 | 0.00 (0.00, 0.16)  0 | 0.00 (0.00, 0.13)  1 | 0.780 |
| Agoraphobia  NA | 0.00 (0.00, 0.00)  1 | 0.00 (0.00, 0.00)  0 | 0.00 (0.00, 0.00)  1 | 1.000 |
| General Phobia  NA | 0.29 (0.00, 0.57)  1 | 0.21 (0.00, 0.43)  0 | 0.29 (0.00, 0.71)  1 | 0.202 |
| Social Phobia  NA | 0.14 (0.00, 0.50)  1 | 0.14 (0.00, 0.32)  0 | 0.14 (0.00, 0.57)  1 | 0.246 |
| Specific Phobia  NA | 0.17 (0.00, 0.50)  1 | 0.17 (0.00, 0.33)  0 | 0.17 (0.00, 0.50)  1 | 0.528 |
| Total Phobia  NA | 0.21 (0.09, 0.38)  1 | 0.19 (0.06, 0.28)  0 | 0.23 (0.10, 0.47)  1 | 0.222 |
| Loss of function  NA | 0.00 (0.00, 0.20)  1 | 0.00 (0.00, 0.05)  0 | 0.00 (0.00, 0.20)  1 | 0.271 |
| Competence  NA | 2.25 (1.88, 2.50)  1 | 2.25 (2.06, 2.50)  0 | 2.13 (1.75, 2.56)  1 | 0.359 |
| Relationship Problems  NA | 2.56 (2.44, 2.78)  1 | 2.67 (2.44, 2.89)  0 | 2.56 (2.33, 2.78)  1 | 0.360 |
| Inventory for Temperament in Children (IKT) for age 2-8 years | | | | |
| Category | Total (n=92) | Control Group  (n=43) | Video Group (n=49) | P value |
| Frustration  NA | 15.0 (5.0, 37.5)  2 | 15.0 (5.0, 35.0)  0 | 15.0 (5.0, 40.0)  2 | 0.867 |
| Inhibition  NA | 45.0 (19.4, 65.0)  3 | 45.0 (20.0, 62.5)  0 | 45.0 (21.3, 70.0)  3 | 0.882 |
| Activity  NA | 40.0 (15.0, 65.0)  2 | 40.0 (8.8, 55.0)  0 | 50.0 (23.8, 75.0)  2 | 0.141 |
| Permanence  NA | 65.0 (37.5, 85.0)  2 | 65.0 (42.5, 85.0)  0 | 65.0 (28.8, 85.0)  2 | 0.468 |
| Sensoric  NA | 35.0 (8.8, 67.5)  2 | 30.0 (6.3, 65.0)  0 | 40.0 (10.0, 70.0)  2 | 0.948 |
| Conduct problems evaluated using the Strengths and Difficulties Questionnaire | | | | |
| Category | Total (n=105) | Control Group (n=51) | Video Group (n=54) | P value |
| Emotional Difficulties  NA | 2.0 (1.0, 3.0)  2 | 1.0 (1.0, 3.0)  0 | 2.0 (1.0, 3.0)  2 | 0.529 |
| Conduct Problems  NA | 2.0 (1.0, 3.0)  2 | 2.0 (1.0, 3.0)  0 | 2.0 (1.0, 4.0)  2 | 0.316 |
| Hyperactivity   NA | 4.0 (2.0, 5.0)  2 | 3.0 (2.0, 5.0)  0 | 4.0 (2.0, 6.3)  2 | 0.064 |
| Peer Problems   NA | 1.0 (0.0, 3.0)  2 | 1.0 (0.0, 2.0)  0 | 1.5 (0.0, 3.3)  2 | 0.222 |
| Prosocial Behaviour  NA | 8.0 (7.0, 9.0)  2 | 8.0 (7.0, 9.0)  0 | 8.0 (6.8, 9.0)  2 | 0.687 |

**Supplementary Table S2.** Patient’s characteristics of children’s personality traits. Values are median (IQR) showing the raw data of their respective raw values for personality traits. * P values for the comparison of video versus control group from Fisher’s exact test (categorical variables) or Wilcoxon rank sum test (continuous variables); NA, not appclibable

| Score, Median | Value at | Total | Control Group | Video Group | P value |
| --- | --- | --- | --- | --- | --- |
| Separation  > 0.3 | N | 35 | 16 | 19 |  |
|  | T3 | 40 (29, 54) | 37 (29, 40) | 41 (31, 60) | 0.369 |
| Separation  <= 0.3 | N | 36 | 20 | 16 |  |
|  | T3 | 34 (33, 47) | 42 (33, 50) | 33 (29, 34) | 0.017 |
| Panic  > 0 | N | 21 | 11 | 10 |  |
|  | T3 | 40 (33, 58) | 35 (31, 40) | 51 (40, 70) | 0.031 |
| Panic  = 0 | N | 50 | 25 | 25 |  |
|  | T3 | 34 (29, 48) | 40 (33, 50) | 33 (27, 40) | 0.018 |
| Agoraphobia  > 0 | N | 13 | 7 | 6 |  |
|  | T3 | 40 (33, 58) | 40 (33, 64) | 42 (40, 5) | 0.719 |
| Agoraphobia  = 0 | N | 58 | 29 | 29 |  |
|  | T3 | 35 (29, 48) | 40 (33, 48) | 33 (29, 44) | 0.211 |
| General Phobia  > 0.286 | N | 30 | 13 | 17 |  |
|  | T3 | 40 (33, 55) | 40 (33, 41) | 41 (33, 62) | 0.365 |
| General Phobia  <= 0.286 | N | 41 | 23 | 18 |  |
|  | T3 | 33 (29, 48) | 40 (33, 50) | 33 (28, 37) | 0.026 |
| Social Phobia  > 0.143 | N | 30 | 13 | 17 |  |
|  | T3 | 40 (32, 49) | 40 (29, 47) | 40 (33, 62) | 0.916 |
| Social Phobia  <= 0.143 | N | 41 | 23 | 18 |  |
|  | T3 | 38 (29, 48) | 40 (33, 49) | 33 (28, 43) | 0.125 |
| Specific Phobia  > 1.67 | N | 31 | 15 | 16 |  |
|  | T3 | 38 (29, 55) | 35 (29, 64) | 39 (29, 50) | 0.952 |
| Specific Phobia  <= 0.167 | N | 40 | 21 | 19 |  |
|  | T3 | 38 (33, 48) | 40 (33, 48) | 33 (30, 47) | 0.252 |
| Total Phobia  > 0.205 | N | 34 | 16 | 18 |  |
|  | T3 | 40 (33, 60) | 39 (32, 48) | 41 (33, 61) | 0.522 |
| Total Phobia  <= 0.205 | N | 37 | 20 | 17 |  |
|  | T3 | 33 (29, 47) | 40 (33, 48) | 33 (27, 33) | 0.037 |
| FL_ANX  > 0 | N | 23 | 9 | 14 |  |
|  | T3 | 40 (33, 60) | 44 (40, 73) | 37 (30, 54) | 0.343 |
| FL_ANX  = 0 | N | 48 | 27 | 21 |  |
|  | T3 | 35 (29, 44) | 38 (31, 46) | 33 (29, 44) | 0.504 |
| Competence  > 2.250 | N | 30 | 16 | 14 |  |
|  | T3 | 38 (3.3, 50) | 37 (32, 48) | 39 (33, 48) | 0.850 |
| Competence  <= 2.250 | N | 41 | 20 | 21 |  |
|  | T3 | 37 (29, 44) | 40 (33, 45) | 33 (29, 44) | 0.213 |

**Supplementary Table S3.** Personality trait ‘anxiety’ as outlined by Döpfner and Görtz-Dorten for age ≥4 years. Scores showing above or below the median values of their respective raw values for personality traits. Allocated to the anxiety score measured with the mYPAS-SF at induction of anesthesia (T3). .Values are median (IQR). P values for the comparison of video versus control group from Fisher’s exact test (categorical variables) or Wilcoxon rank sum test (continuous variables)

| Score, median | Value at | Total | Control Group | Video Group | P value |
| --- | --- | --- | --- | --- | --- |
| Frustration  > 15 | N | 44 | 22 | 22 |  |
|  | T3 | 40 (38, 63) | 40 (36, 69) | 41 (38, 60) | 0.524 |
| Frustration  <= 15 | N | 46 | 21 | 25 |  |
|  | T3 | 38 (33, 49) | 44 (35, 50) | 33 (29, 44) | 0.137 |
| Inhibition  > 50 | N | 41 | 19 | 22 |  |
|  | T3 | 40 (35, 53) | 40 (38, 48) | 38 (33, 57) | 0.318 |
| Inhibition  <= 50 | N | 48 | 24 | 24 |  |
|  | T3 | 40 (33, 55) | 40 (33, 57) | 39 (33, 52) | 0.893 |
| Activity  > 47.5 | N | 45 | 18 | 27 |  |
|  | T3 | 40 (33, 62) | 40 (36, 58) | 38 (33, 64) | 0.676 |
| Activity  <= 47.5 | N | 45 | 25 | 20 |  |
|  | T3 | 40 (33, 47) | 40 (35, 50) | 37 (33, 44) | 0.377 |
| Persistance > 70 | N | 41 | 18 | 23 |  |
|  | T3 | 38 (34, 44) | 39 (35, 44) | 38 (34, 48) | 0.624 |
| Persistance  <= 70 | N | 49 | 25 | 24 |  |
|  | T3 | 41 (33, 67) | 44 (38, 80) | 38 (29, 63) | 0.196 |
| Sensoric  > 45 | N | 40 | 20 | 20 |  |
|  | T3 | 42 (37, 63) | 42 (38, 60) | 42 (34, 63) | 0.903 |
| Sensoric  <= 45 | N | 50 | 23 | 27 |  |
|  | T3 | 38 (33, 46) | 40 (33, 50) | 38 (32, 44) | 0.374 |

**Supplementary Table S4.** Inventory for Temperament in Children (IKT) for age 2-8 years. Scores showing above or below the median values of their respective raw values for the personality traits. Allocated to the anxiety score measured with the mYPAS-SF at induction of anesthesia (T3). Values are median (IQR). P values for the comparison of video versus control group from Fisher’s exact test (categorical variables) or Wilcoxon rank sum test (continuous variables)

| Score, median | Value at | Total | Control Group | Video Group | P value |
| --- | --- | --- | --- | --- | --- |
| Emotional Difficulties  >2 | N | 32 | 15 | 17 |  |
|  | T3 | 42 (33, 63) | 40 (34, 52) | 44 (33, 67) | 0.777 |
| Emotional Difficulties <=2 | N | 71 | 36 | 35 |  |
|  | T3 | 38 (33, 45) | 40 (33, 50) | 38 (33, 42) | 0.164 |
| Conduct problems  > 2 | N | 37 | 18 | 19 |  |
|  | T3 | 40 (33, 62) | 40 (34, 78) | 41 (35, 57) | 0.573 |
| Conduct problems  <= 2 | N | 66 | 33 | 33 |  |
|  | T3 | 38 (33, 46) | 40 (33, 47) | 38 (33, 44) | 0.347 |
| Hyperactivity  > 4 | N | 39 | 17 | 22 |  |
|  | T3 | 40 (33, 58) | 44 (38, 83) | 38 (32, 46) | 0.122 |
| Hyperactivity  <= 4 | N | 64 | 34 | 30 |  |
|  | T3 | 38 (33, 47) | 40 (33, 44) | 38 (33, 56) | 0.930 |
| Problems with peer group  > 1 | N | 46 | 20 | 26 |  |
|  | T3 | 38 (30, 44) | 38 (33, 45) | 38 (29, 43) | 0.435 |
| Problems with peer group  <= 1 | N | 57 | 31 | 26 |  |
|  | T3 | 41 (33, 55) | 44 (33, 53) | 40 (33, 56) | 0.676 |
| Prosocial behaviour  > 8 | N | 42 | 21 | 21 |  |
|  | T3 | 38 (33, 50) | 40 (33, 50) | 38 (33, 50) | 0.449 |
| Prosocial behaviour  <= 8 | N | 61 | 30 | 31 |  |
|  | T3 | 40 (33, 50) | 40 (33, 50) | 38 (33, 50) | 0.524 |

**Supplementary Table S5.** Conduct problems evaluated using the Strengths and Difficulties Questionnaire. Scores showing above or below the median values of their respective raw values for the personality traits. Allocated to the anxiety score measured with the mYPAS-SF at induction of anesthesia (T3). Values are median (IQR). P values for the comparison of video versus control group from Fisher’s exact test (categorical variables) or Wilcoxon rank sum test (continuous variables)

| **Subgroup 2-6 years old children** | | | | |
| --- | --- | --- | --- | --- |
| **Variable** | **Total  (n=41)** | **Control Group (n=20)** | **Video Group (n=21)** | **P value** |
| **T1** | 35 (23, 46) | 34 (23, 44) | 36 (26, 50) | 0.493 |
| **T2** | 36 (31, 46) | 36 (27, 41) | 38 (32, 50) | 0.601 |
| **T3** | 44 (38, 67) | 44 (38, 72) | 41 8 (38, 56) | 0.792 |
| **T2-T1** | 0 (-8, 13) | 0 (-4, 14) | 3 (-8, 14) | 0.937 |
| **T3-T2** | 7 (0, 20) | 10 (1, 21) | 6 (0, 17) | 0.396 |
| **Subgroup 7-10 years old children** | | | | |
| **Variable** | **Total  (n=64)** | **Control Group (n=31)** | **Video Group (n=33)** | **P value** |
| **T1** | 29 (23, 38) | 29 (23, 39) | 33 (23, 35) | 0.471 |
| **T2** | 35 (29, 44) | 35 (29, 42) | 35 (27, 44) | 0.696 |
| **T3** | 36 (29, 44) | 40 (33, 45) | 33 (29, 44) | 0.251 |
| **T2-T1** | 4 (-1, 10) | 3 (-2, 10) | 4 (0, 11) | 0.522 |
| **T3-T2** | 0 (-3, 6) | 1 (-3, 12) | 0 (-6, 6) | 0.348 |

**Supplementary Table S6.** Anxiety scores measured with the mYPAS-SF. Values are median (IQR). P values for the comparison of video versus control group from Fisher’s exact test (categorical variables) or Wilcoxon rank sum test (continuous variables). T1 = at the ward, T2 = while transfer to theatre, T3 = during induction of anesthesia, T3-T2 and T3-T1 T3-T2 show the difference between the 2 timepoints (negative values indicating decrease, positive values indicating increase of anxiety).
